# Antennal transcriptome analysis of chemosensory genes in the cowpea beetle, *Callosobruchus maculatus* (F.)

**DOI:** 10.1371/journal.pone.0262817

**Published:** 2022-01-19

**Authors:** Keisuke Tanaka, Kenji Shimomura, Akito Hosoi, Yui Sato, Yukari Oikawa, Yuma Seino, Takuto Kuribara, Shunsuke Yajima, Motohiro Tomizawa

**Affiliations:** 1 NODAI Genome Research Center, Tokyo University of Agriculture, Setagaya-ku, Tokyo, Japan; 2 Department of Chemistry for Life Sciences and Agriculture, Tokyo University of Agriculture, Setagaya-ku, Tokyo, Japan; 3 Department of Bioscience, Tokyo University of Agriculture, Setagaya-ku, Tokyo, Japan; Universita degli Studi della Basilicata, ITALY

## Abstract

Olfaction, one of the most important sensory systems governing insect behavior, is a possible target for pest management. Therefore, in this study, we analyzed the antennal transcriptome of the cowpea beetle, *Callosobruchus maculatus* (F.) (Coleoptera: Chrysomelidae: Bruchinae), which is a major pest of stored pulses and legumes. The *de novo* antennal RNA-seq assembly results identified 17 odorant, 2 gustatory, and 10 ionotropic receptors, 1 sensory neuron membrane protein, and 12 odorant-binding and 7 chemosensory proteins. Moreover, differential gene expression analysis of virgin male and female antennal samples followed by qRT-PCR revealed 1 upregulated and 4 downregulated odorant receptors in males. We also performed homology searches using the coding sequences built from previously proposed amino acid sequences derived from genomic data and identified additional chemosensory-related genes.

## Introduction

Insects use olfactory signals in many behavioral contexts, such as locating food, mating, identifying oviposition sites, and escaping predators [1]. To detect olfactory signals, insects have developed a sensory system consisting of olfactory receptor neurons (ORNs) housed in the hair sensilla on the antennae and maxillary palps [2]. There are three chemoreceptor gene families in insects: odorant (OR), gustatory (GR), and ionotropic receptor (IR) families. Insect ORs contain seven transmembrane domains and have a membrane topology with intracellular N-termini and extracellular C-termini, opposite to that of G-protein coupled receptors mediating chemoperception in many vertebrates [3, 4]. GRs have a similar membrane topology to that of ORs [5], and IRs comprise three transmembrane domains with an extracellular N-terminus and a cytoplasmic C-terminus [6, 7]. Additionally, other gene families encode proteins that have crucial roles in olfaction, including odorant-binding proteins (OBPs), chemosensory proteins (CSPs), and sensory neuron membrane proteins (SNMPs) [8, 9]. Insect OBPs and CSPs are soluble proteins with an N-terminal signal peptide that is removed during processing and consist of 130–150 and 100–120 amino acid residues, respectively [10, 11]. Based on the number of cysteine residues, OBPs are classified into classical OBPs (6 cysteine residues with 3 disulfide bonds) and non-classic OBPs, including plus-C OBPs (8 cysteine residues and a conserved proline), minus-C OBPs (4–5 conserved cysteine residues lacking C2 and C5 cysteines), dimer OBPs (12 conserved cysteines), and atypical OBPs (9–10 conserved cysteine residues and a long C-terminus) [12, 13]. The OBP motif in coleopteran species is conserved (C1-X_21-68_-C2-X_3_-C3-X_21-46_-C4-X_8-28_-C5-X_8-9_-C6, where X represents any amino acid) [14]. In the case of CSPs, that contain four cysteine residues with a highly conserved pattern (C1-X_6-8_-C2-X_18_-C3-X_2_-C4) [14]. SNMPs belong to a gene family of human protein CD36, which contains two transmembrane domains and a large extracellular loop with several cysteines [15–17].

Generally, odorant molecules enter the sensilla through pores [18] and are then transported through the lymph via OBPs or CSPs to the ORN membrane [19], where they interact with ORs or IRs, triggering an action potential [20, 21]. The SNMPs also play a role in pheromone perception [22].

To date, in the applied development of sustainable pest management, functional characterization of chemosensory-based detection of ligands, such as pheromones and host odors, has been studied, especially of pests. The order Coleoptera is the largest in the animal kingdom with approximately 390,000 described species, and it contains economically important agricultural pests [23]. Within this order, *Tribolium castaneum* (Herbst) was the first species to have its genome sequenced [24]. To date, an additional two coleopteran species have been sequenced, namely, *Anoplophora glabripennis* Motschulsky [25] and *Leptinotarsa decemlineata* Say, which was the first example of a chrysomelid beetle [26]. Alternatively, antennal transcriptomic analyses have been performed (e.g. [27–29]). For example, chemosensory gene families have been identified in various chrysomelid beetles, including *Colaphellus bowringi* Baly [30], *L*. *decemlineata* [31], *Ambrostoma quadriimpressum* Motschulsky [32], *Phyllotreta striolata* (F.) [33], *Pyrrhalta maculicollis* (Mots.), *P*. *aenescens* Fairmaire [34], and *Ophraella communa* LeSage [35]. Moreover, RNA sequences (RNA-seq) of antennal OBPs and CSPs of the southern cowpea beetle, *Callosobruchus chinensis* L., have also been analyzed [36], indicating that these analyses are common practice.

The cowpea beetle, *Callosobruchus maculatus* (F.) (Coleoptera: Chrysomelidae: Bruchinae), is an important pest of stored legumes, particularly of cowpea, *Vigna unguiculata* (L.) Walp., an important grain legume distributed worldwide [37]. The grain loss during cowpea storage is estimated to be approximately 100% owing to the perforations in grains after beetle emergence [38], thus decreasing seed quality and yield. Regarding the semiochemical-based communication of *C*. *maculatus*, five short-chain fatty acids, including (*Z*)- and (*E*)-3-methyl-2-heptenoic acid, (*Z*)- and (*E*)-3-methyl-3-heptenoic acid, and 3-methyleneheptanoic acid, have been identified as female-produced sex-attractant pheromones [39]. During the mating process, the species also produces short-range contact sex pheromones, such as 2,6-dimethyloctanedioic acid and methyl-branched C_27_–C_35_ straight-chain synergistic hydrocarbons, to elicit courtship behavior and copulation [40]. In addition, the contact sex pheromone analogs, 2-methyloctanedioic acid, 3-methyloctanedioic acid, and nonanedioic acid, produced by the congeneric species, *Callosobruchus rhodesianus* (Pic.), also result in copulatory behavior in male *C*. *maculatus* [41]. Female *C*. *maculatus* are attracted within a short range to the surface wax of legume seeds, C_15_–C_32_ n-alkanes, and seed volatiles, such as 3-octanone, 3-octanol, linalool oxide, 1-octanol, and nonanal, suggesting that females can locate host legumes for oviposition [42, 43]. However, the molecular basis of chemical perception, including pheromone perception, remains unknown; however, transcriptional analyses have been performed following the genome assembly of *C*. *maculatus* [44–46]. Furthermore, information on annotated chemosensory genes is still limited, possibly because previous studies analyzed the transcriptomes of the abdomen, head, and thorax to understand the digestive and reproductive gene expression profiles of *C*. *maculatus* [44, 45]. Therefore, in the present study, we focused on *de novo* RNA-seq to analyze the antennal transcriptome of the species and conducted homology searches to identify chemosensory-related genes for coding sequences (CDSs), which were derived from the genomic data of *C*. *maculatus* but still not annotated. Additionally, we carried out phylogenetic analyses using the annotated chemosensory genes from genomically analyzed coleopteran beetle data. Finally, we compared the expressions of differentially expressed genes (DEGs) in the antennae of virgin *C*. *maculatus* males and females.

## Methods

### Insect rearing

Laboratory colonies of *C*. *maculatus* were used for the study. The insects were reared on *Vigna angularis* (Willd.) Ohwi and Ohashi in a plastic container at 28°C in a dark incubator. The adults that emerged from the beans were immediately separated by sex, anesthetized on ice, and had their antennae excised and stored at -80°C until further use.

### RNA extraction, cDNA library construction, and Illumina sequencing

Sixty antennae from virgin male and female *C*. *maculatus* were crushed using a BioMasher II (Nippi Inc., Tokyo, Japan). Thereafter, total RNA was extracted using the ReliaPrep RNA Cell Miniprep System (Promega Corporation, Madison, WI, USA) following the manufacturer’s protocol. RNA quality was confirmed based on an RNA Integrity Number >8 using an Agilent RNA 6000 Nano Kit in an Agilent 2100 Bioanalyzer (Agilent Technologies, Santa Clara, CA, USA). Afterward, a cDNA library was prepared using a TruSeq RNA Library Preparation Kit v2 (Illumina, San Diego, CA, USA) using 100 ng of total RNA according to the manufacturer’s protocol. The library was sequenced on the Illumina sequencing platform (Illumina HiSeq 2500), and 100 bp paired-end reads were generated. The read data were deposited in the DDBJ Sequence Read Archive (DRA011785).

### *de novo* RNA-Seq assembly

Raw reads were adapter-trimmed and quality-filtered using fastp version 0.20.0 [47]. The trimmed and filtered reads from each sample were assembled *de novo* using Trinity version 2.8.5 [48] with default parameters. The CDSs were predicted using TransDecoder version 5.3.0 (https://github.com/TransDecoder/TransDecoder/wiki), and those with approximately 98% similarity were clustered using the cd-hit-est program of CD-HIT version 4.8.1 [49]. The clustered CDS contigs were defined as unigenes in this study. The unigene sequences were evaluated using benchmarking universal single-copy orthologs (BUSCO) analysis version 3.0.2 [50]. In addition, the unigenes were annotated with e-value <1e^-5^ using the NCBI non-redundant database and the blastx program [51].

### Phylogenetic analyses of the *in silico* predicted chemosensory system

To narrow down chemosensory-related genes from the unigenes, an insect antennal dataset was customized from the NCBI protein database using the following keywords: "odorant receptor," "gustatory receptor," "sensory neuron membrane protein," "ionotropic receptor," "odorant-binding protein," and "chemosensory protein." The unigenes were then translated into amino acid sequences and annotated using the blastp program against the custom dataset [51]. In addition, a dataset of amino acid sequences obtained from the *C*. *maculatus* genome (GCA_900659725.1_ASM90065972v1) from the NCBI Genome database (https://www.ncbi.nlm.nih.gov/genome) was also annotated using the same protocol.

The candidate chemosensory proteins (ORs, GRs, IRs, and SNMPs) were predicted using the TMHMM server 2.0 (http://www.cbs.dtu.dk/services/TMHMM/). Among them, the helical domains of ORs and GRs comprising seven helices with topology from inside to outside, IRs comprising three helices, SNMPs comprising two transmembrane helices with intracellular N- and C-termini, and eight cysteine residues were localized on the extracellular domain. The candidate OBPs and CSPs with Sec signal peptides were predicted using the signalP 5.0 server (http://www.cbs.dtu.dk/services/SignalP/).

Amino acid sequences for each protein, consisting of both the candidate protein with transmembrane domains or signal peptide and the target protein identified using the BLAST search, were aligned using MAFFT version 7.214 [52] with default parameters. Moreover, maximum-likelihood (ML) phylogenetic analysis for the aligned sequence was performed using IQ-TREE multicore version 1.6.12 with the best-fit model in ModelFinder and 1,000 ultra-fast bootstrap replicates [53]. The ML phylogenetic trees were drawn using CLC Genomics Workbench 20 (Qiagen, Germantown, MD, USA). Amino acid sequences of chemosensory-related proteins from other insects were also obtained to estimate the *C*. *maculatus* candidate proteins. Accordingly, OR sequences of *T*. *castaneum*, *A*. *glabripennis*, and *L*. *decemlineata* were obtained from Mitchell et al. [54]. *Leptinotarsa decemlineata* GR sequences, *T*. *castaneum*, and *L*. *decemlineata* IR sequences were obtained from Schoville et al. [26]. Furthermore, SNMP sequences for *A*. *planipennis*, *A*. *glabripennis*, and *D*. *ponderosae* were retrieved from Andersson et al. [55], and those for *T*. *castaneum* were obtained from Dippel et al. [56]. In addition, OBP and CSP sequences for *T*. *castaneum* were obtained from Dippel et al. [57], and OBP and CSP sequences for *C*. *chinensis* were retrieved from Zhang et al. [36], and OBP sequences for *L*. *decemlineata* were obtained from Schoville et al. [26]. OBP and CSP sequences for *A*. *glabripennis* were obtained from Wang et al. [58] and Andersson et al. [55], respectively.

### Identification of DEGs

The trimmed and filtered read data were mapped onto the *de novo* assembled unigenes using CLC Genomics Workbench 20 with the following parameters: mismatch cost = 2, insertion cost = 3, deletion cost = 3, length fraction = 0.8, and similarity fraction = 0.8. After statistical analysis based on a generalized linear model, DEGs with a change more than |2|-fold and false discovery rate (FDR)-adjusted *p*-value < 0.05 were selected. Gene ontology (GO) annotation and enrichment analysis of the DEGs were conducted using Blast2GO Basic version 6.0.3 [59].

### qRT-PCR

The total RNA was quantified using a Nanodrop spectrophotometer (Thermo Fisher Scientific, Waltham, MA, USA), and first-strand cDNA was synthesized using a ReverTra Ace qPCR RT kit (Toyobo, Osaka, Japan) according to the manufacturer’s protocol. Gene-specific primers (listed in S1 Table) were designed using Primer3.

qRT-PCR reactions were carried out in a StepOnePlus Real-Time PCR System (Applied Biosystems Inc., Foster City, CA, USA) using THUNDERBIRD Next SYBR qPCR Mix (Toyobo) with an amplification step (95°C for 30 s, followed by 45 cycles of 95°C for 5 s and 60°C for 30 s), followed by a dissociation step (a cycle of 95°C for 15 s, 60°C for 30 s, and 95°C for 15 s) for melting curve analysis. The expression levels were calculated using the ΔΔCt method [60]. The mean Ct value for each gene was calculated using three replicates, and the glyceraldehyde-3-phosphate dehydrogenase gene was used to normalize gene expression. The relative expression levels of each C. maculatus OR (CmacOR) by sex were compared using a two-tailed Student’s *t*-test. All statistical analyses were performed in R software.

## Results

### Sequencing and *de novo* assembly

The number of raw paired-end reads ranged from 2 × 19,324,335 to 2 × 22,898,011 in each sample, with approximately 98.9–99.0% reads generated as clean sequence reads. A total of 23,840 contigs were identified as unigenes belonging to eukaryotes, arthropods, and insects with BUSCO scores of 94.7, 92.7, and 91.8%, respectively. In addition, 20,024 (84.0%) contigs were functionally annotated using BLAST.

### Identification of chemosensory-related genes

Unigene data and datasets of amino acid sequences derived from *C*. *maculatus* genomic data were functionally characterized into six chemosensory-related gene sets using the customized insect antennal dataset (S2–S7 Tables).

#### ORs

Transmembrane topology prediction identified 26 genes encoding putative ORs (17 from the antennal transcriptomes and 9 from the genomic analysis; CmacOR), with lengths ranging from 335 to 479 amino acid residues.

**Fig 1:**
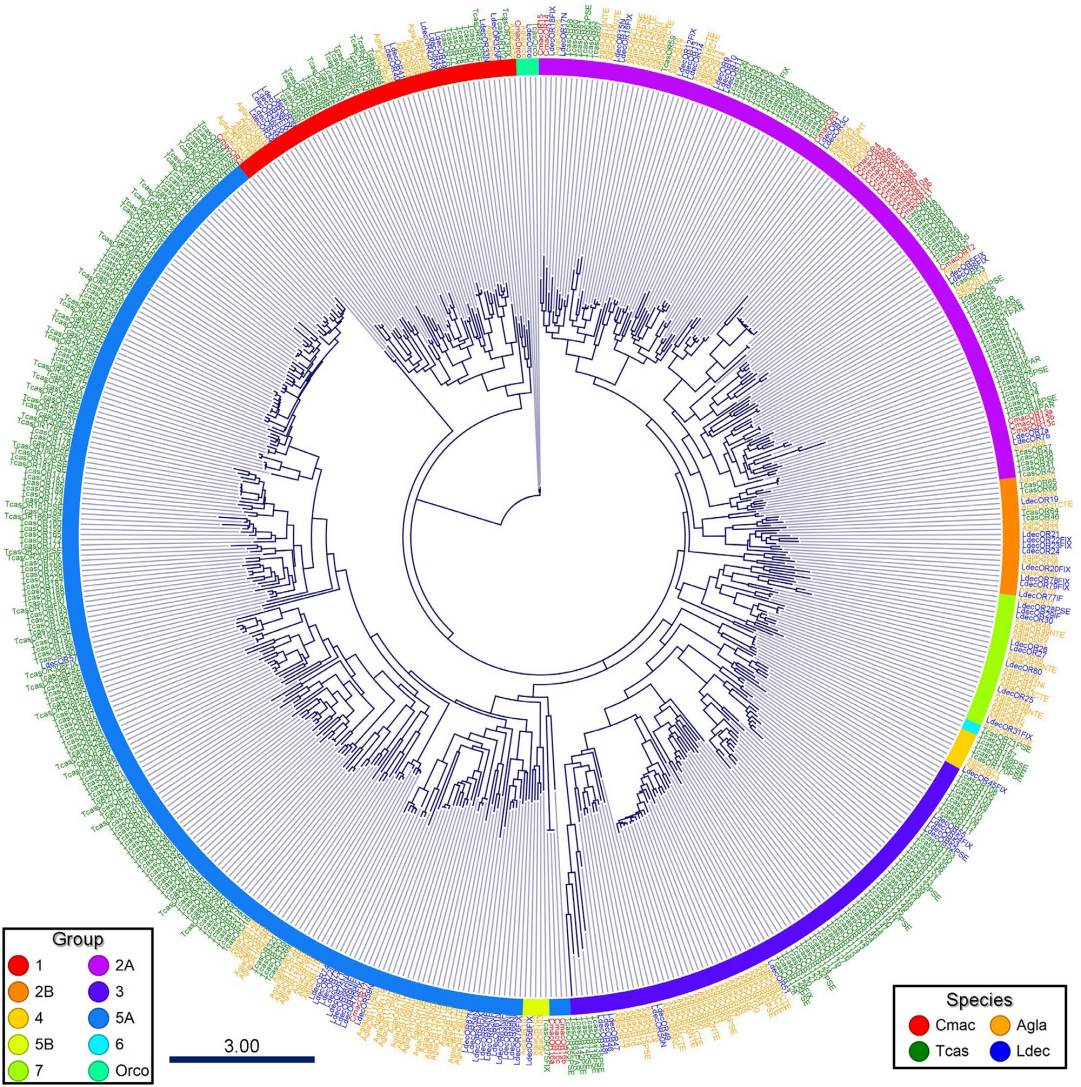
Maximum-likelihood phylogenetic tree of putative *Callosobruchus maculatus* odorant receptors (CmacORs) with insect OR sequences from *Anoplophora glabripennis* (Agla), *Leptinotarsa decemlineata* (Ldec), and *Tribolium castaneum* (Tcas). Thick nodes are supported by a bootstrap value >60%. The rate of amino-acid substitutions per site is shown in the scale bar.

In Coleoptera, phylogenetic analysis revealed that the OR genes were separated into nine major subfamilies [54]; CmacOR gene numbers were allocated based on these subfamilies. The ML phylogenetic tree with genomically identified ORs of three coleopteran species revealed that 20 of 26 CmacORs were clustered around group 2A and followed by group 5A (4) (Fig 1). Within group 2A, a species-specific cluster comprised CmacOR4–11. Only one gene (CmacOR2) was housed in group 1, and no other genes were detected in the other groups.

We identified a putative OR-coreceptor (Orco) gene, namely CmacOrco, which was clustered with the Orco family with a high level of conservation (84–89% amino acid sequence identity) across the three beetles (*A*. *glabripennis*, *L*. *decemlineata*, and *T*. *castaneum*). The CmacOrco gene also showed the highest expression level according to the TPM value (S2 Table).

#### GRs

**Fig 2:**
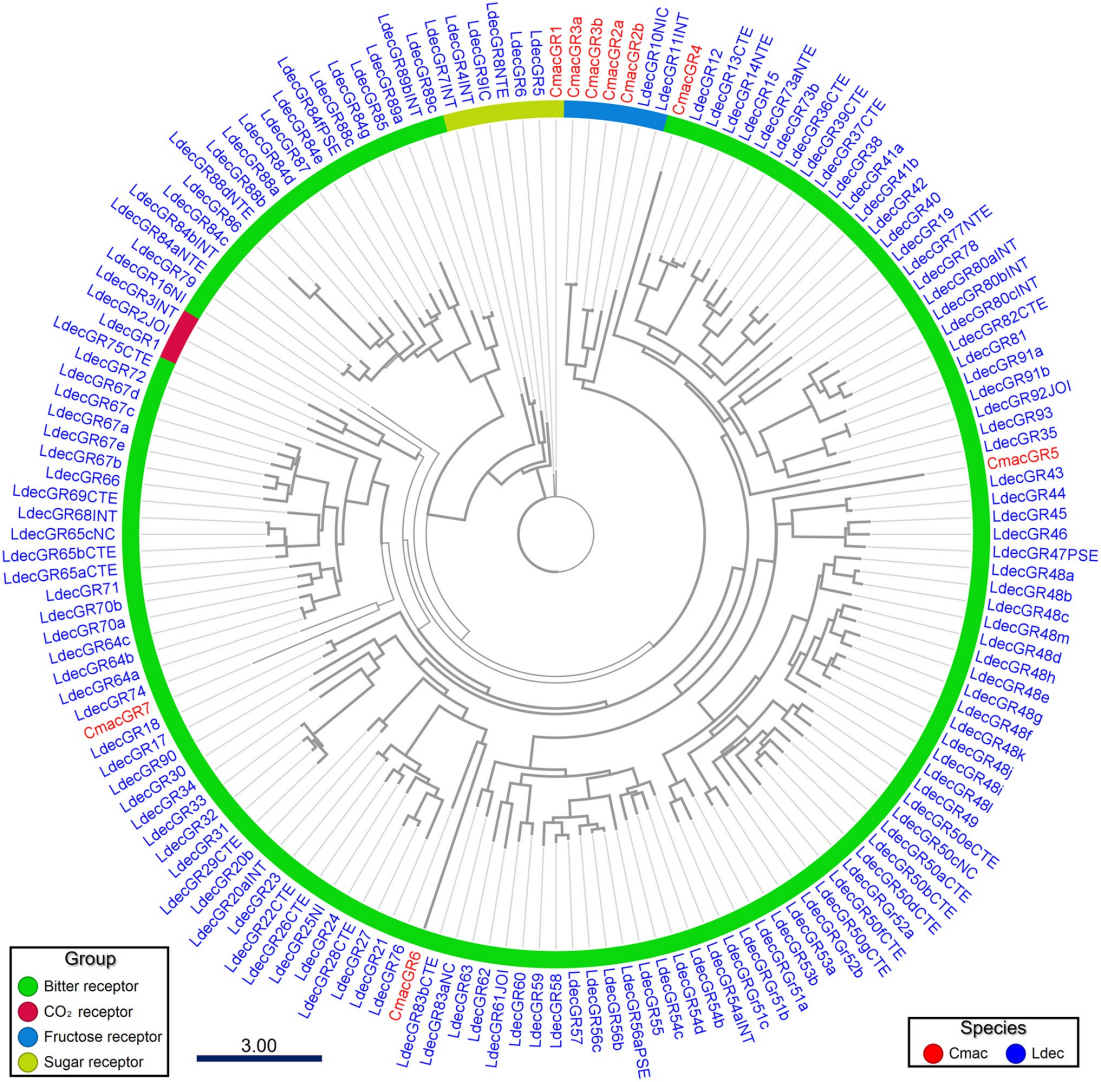
Maximum-likelihood phylogenetic tree of putative *Callosobruchus maculatus* gustatory receptors (GRs) with *Leptinotarsa decemlineata* (Ldec) GR sequences. Thick nodes are supported by a bootstrap value >60%. The rate of amino-acid substitutions per site is shown at the scale bar.

GR-transmembrane topology prediction revealed 2 GRs predicted from the antennal transcriptome, and 7 GRs were annotated upon genomic analysis, namely CmacGR1–7, with intact open reading frames ranging from 330 to 400 amino acid residues. The ML tree constructed using genomically identified *L*. *decemlineata* GRs revealed one sugar receptor (CmacGR1) in the cladogram containing LdecGR4–9 [26] (Fig 2). Additionally, four CmacGRs (CmacGR2a–3b) were clustered with LdecGR10NIC, which was identified as a fructose receptor. The remaining four CmacGRs derived from the genomic data were separately clustered in the bitter group [61].

#### IRs

**Fig 3:**
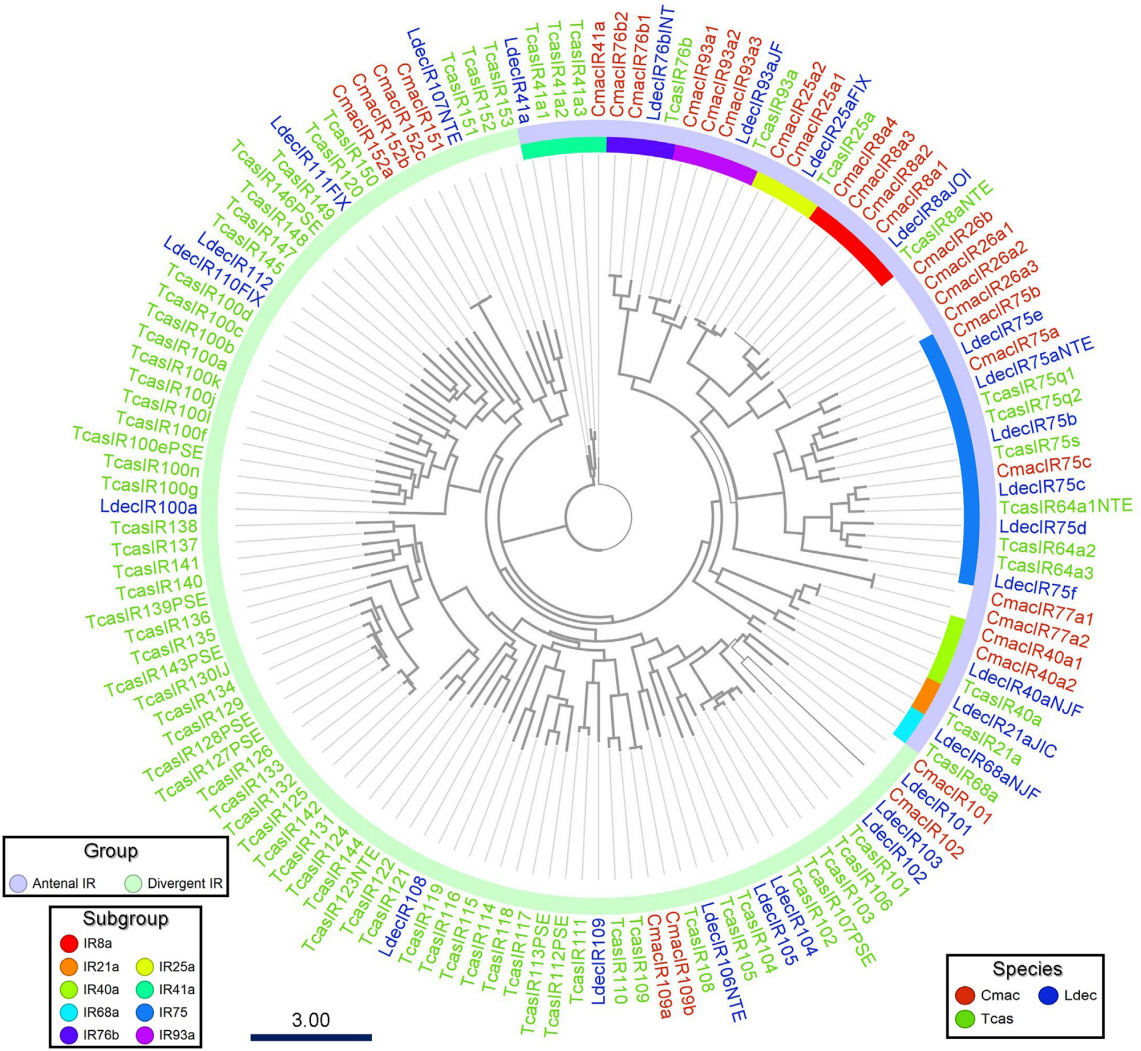
Maximum-likelihood phylogenetic tree of putative *Callosobruchus maculatus* ionotropic receptors (IRs) with *Leptinotarsa decemlineata* (Ldec) and *Tribolium castaneum* (Tcas) IR sequences. Thick nodes are supported by a bootstrap value >60%. The rate of amino-acid substitutions per site is shown in the scale bar.

Transmembrane topology prediction identified 10 IRs from the antennal transcriptome and 21 IRs from the genomic analysis (CmacIRs), ranging from 88 to 927 amino acid residues in length, correspondingly named for their putative *T*. *castaneum* and *L*. *decemlineata* homologs. The ML tree of CmacIRs revealed 23 widely conserved antennal IRs and 8 divergent IRs (Fig 3). Four conserved-receptor-homologs, namely IR8a, IR25a, IR76b, and IR93a, required as co-receptors with the other IRs [62], were detected in the antennal IRs. We also identified the antennal IR41a and IR75 groups, which functioned as olfactory detectors of acids and amines [63–65], and IR40a, which detected humidity [66, 67]. Eight homologs were identified among the divergent IRs (one from the antennal transcript and seven from the genomic annotations).

#### SNMPs

**Fig 4:**
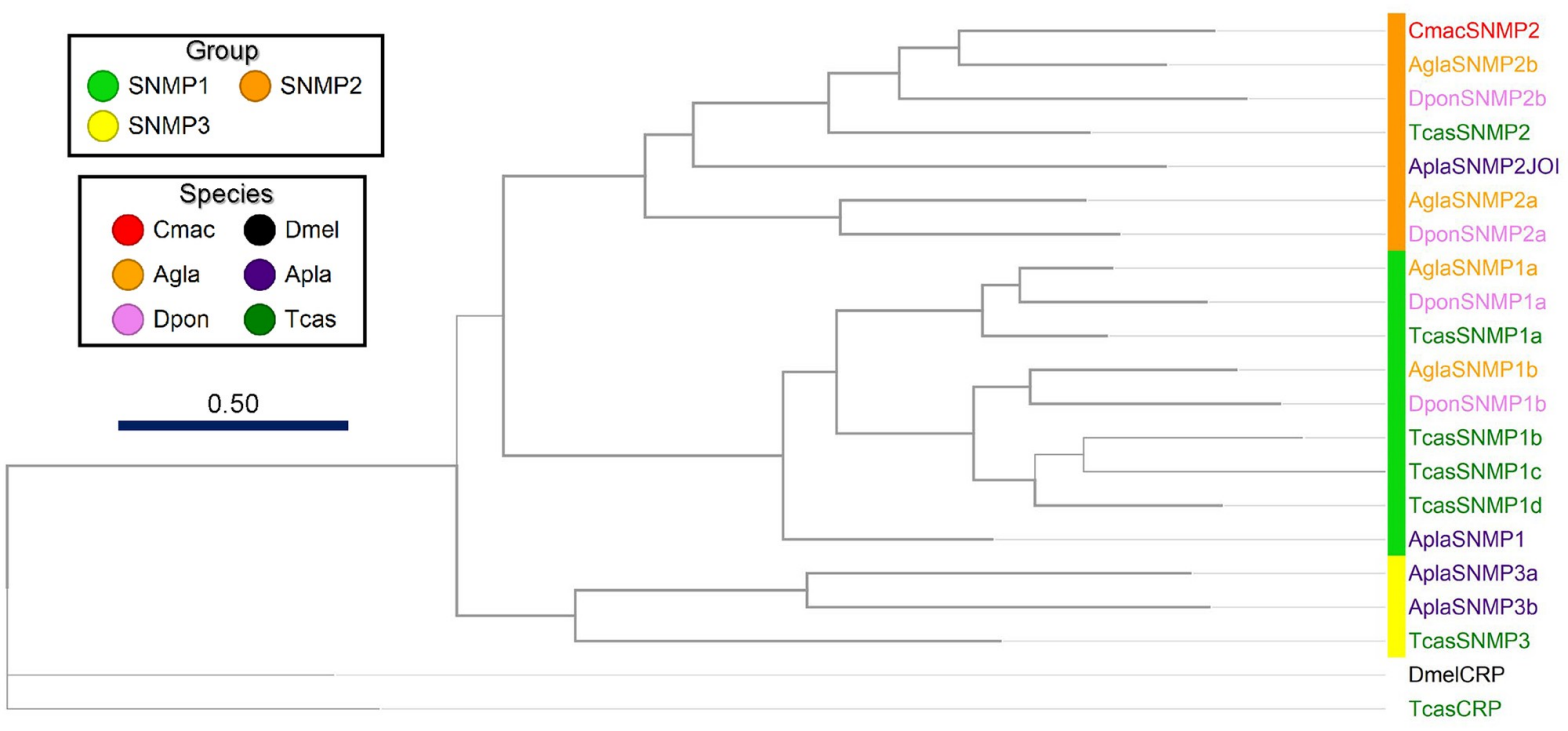
Maximum-likelihood phylogenetic tree of putative *Callosobruchus maculatus* sensory neuron membrane protein (SNMP) with insect SNMP sequences, *Dendroctonus ponderosae* (Dpon), *Agrilus planipennis* (Apla), *Anoplophora glabripennis* (Agla), *Tribolium castaneum* (Tcas), and *Drosophila melanogaster* (Dmel). The tree was rooted with the croquemort (Crq) protein lineage, a member of the CD36 family that is non-SNMP. Thick nodes are supported by a bootstrap value >60%. The rate of amino-acid substitutions per site is shown in the scale bar.

Based on transmembrane topology prediction, we identified one SNMP containing 522 amino acid residues, and the ML tree among coleopteran beetle SNMPs revealed that the gene contained an SNMP2 clade, namely CmacSNMP2 (Fig 4). Although coleopteran SNMPs were categorized into four groups [68], no other SNMPs were detected in this study.

#### OBPs and CSPs

Using signal peptide prediction and motif analysis, 12 OBP genes from the antennal transcriptome and 14 OBP genes from the genomic analysis were identified, with 4 OBPs in common, whose sizes ranged from 118 to 361 amino acid residues. The number of putative OBPs was almost comparable to that in the congeneric species, *C*. *chinensis* (21) [36], correspondingly named for their putative *C*. *chinensis* homologs. Moreover, no plus-C, dimer, and atypical OBPs were identified in this study, while 15 classical and 11 minus-C OBPs were deduced from the 26 predicted CmacOBPs.

**Fig 5:**
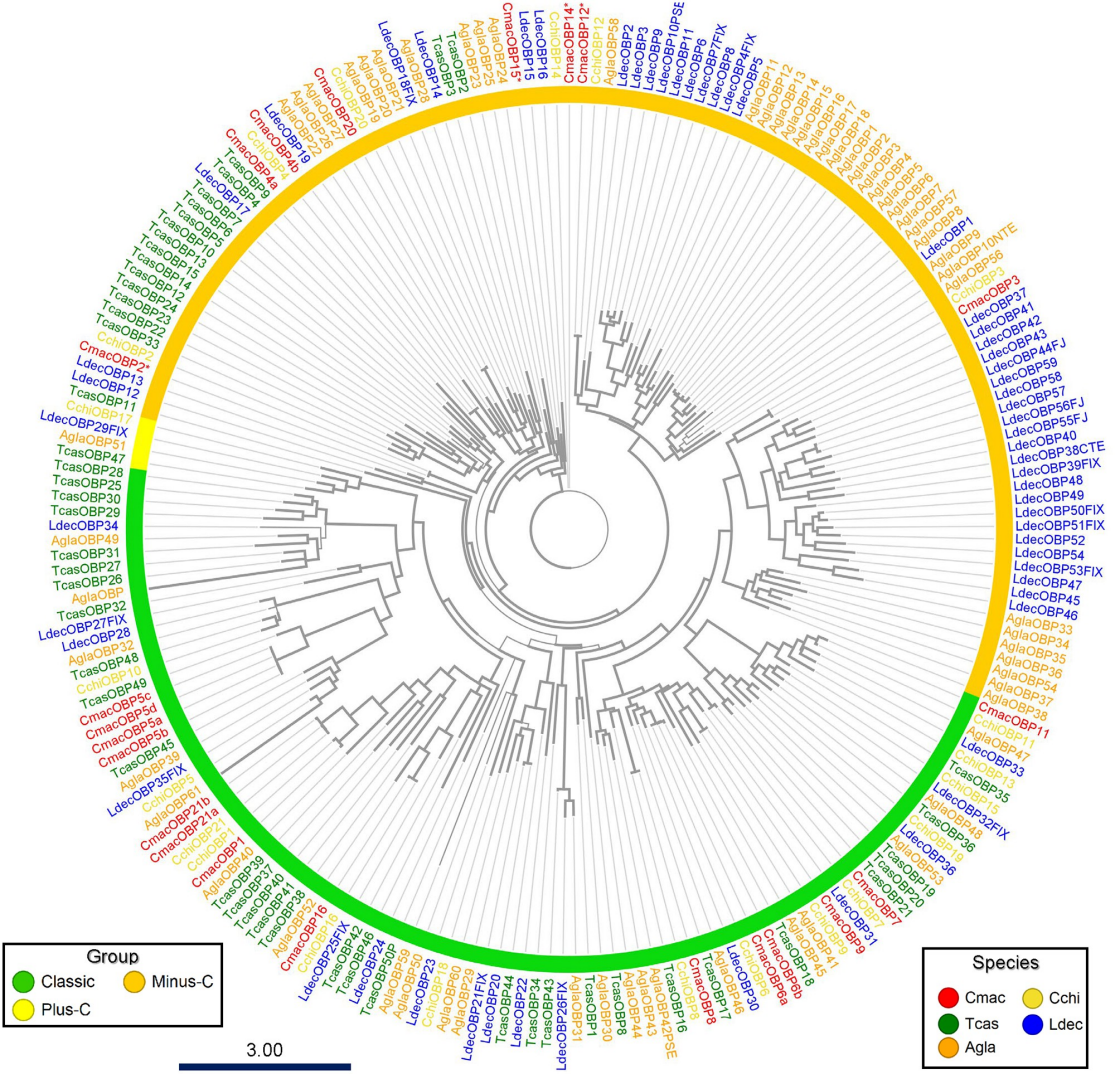
Maximum-likelihood phylogenetic tree of putative *Callosobruchus maculatus* odorant-binding proteins (OBPs) with insect OBP sequences from *Anoplophora glabripennis* (Agla), *Leptinotarsa decemlineata* (Ldec), *Tribolium castaneum* (Tcas), and *Callosobruchus chinensis* (Cchi). Thick nodes are supported by a bootstrap value >60%. The rate of amino-acid substitutions per site is shown in the scale bar. The four CmacOBPs suffixed with asterisks were a perfect match between the antennal transcriptome and genomic analyses.

The ML tree revealed 14 orthologous pairs between *C*. *maculatus* and *C*. *chinensis*; among them, almost all the pairs shared 93–100% identical residues. Although some CchiOBPs lack N-terminus amino acids, only CmacOBP11 and CchiOBP11 shared 70% identical residues (Fig 5). Six OBPs were specific to *C*. *maculatus*. Some CchiOBPs were grouped in different clusters from those previously reported; CchiOBP18 was in the classic OBP and CchiOBP17 was in the plus-C clade, possibly due to the different tree construction methods [36].

The minus-C CmacOBPs clustered into two subfamilies in the tree; CmacOBP2, CmacOBP4a, 4b, CmacOBP12, CmacOBP14, CmacOBP15, and CmacOBP20 clustered with several minus-C OBPs from *T*. *castaneum*, *A*. *glabripennis*, and *L*. *decemlineata*, and the remaining CmacOBP3 clustered with the other subfamily comprising the species-specific lineage expansion of *A*. *glanbripennis* and *L*. *decemilineata* [54].

**Fig 6:**
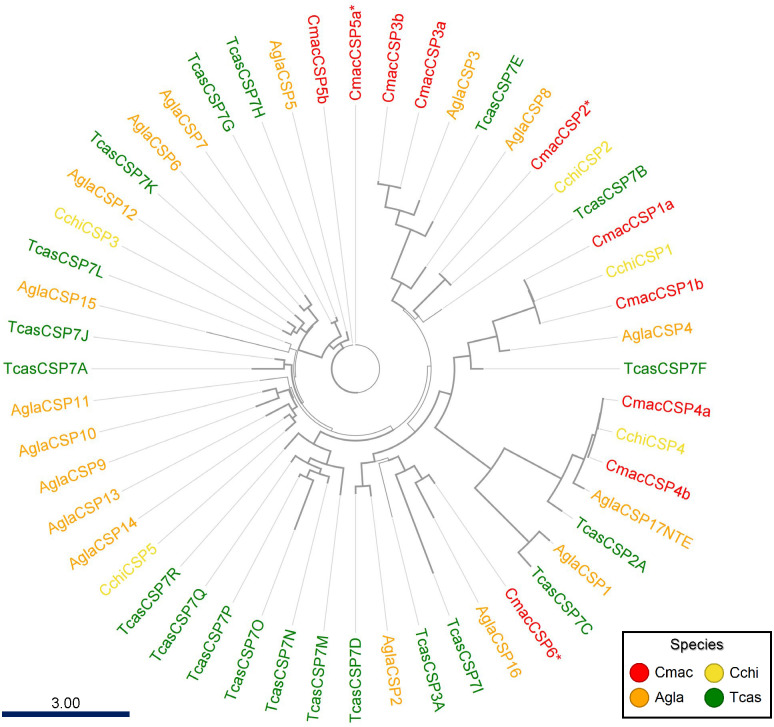
Maximum-likelihood phylogenetic tree of putative *Callosobruchus maculatus* chemosensory proteins (CSPs) with insect CSP sequences from *Anoplophora glabripennis* (Agla), *Tribolium castaneum* (Tcas), and *Callosobruchus chinensis* (Cchi). Thick nodes are supported by a bootstrap value >60%. The rate of amino-acid substitutions per site is shown in the scale bar. The three CmacCSPs suffixed with asterisks were a perfect match between the antennal transcriptome and genomic analyses.

The antennal transcriptome and genomic analysis revealed 7 and 6 CSP genes (117–317 amino acids long), respectively, with 3 CSPs in common, and the names were designated CmacCSP1–6. The phylogenetic ML tree analysis showed three orthologous pairs between *C*. *maculatus* and *C*. *chinensis*, sharing 98–99% identical residues, and three CSPs were specific to *C*. *maculatus* (Fig 6).

### Detection of DEGs and qRT-PCR analyses

**Fig 7:**
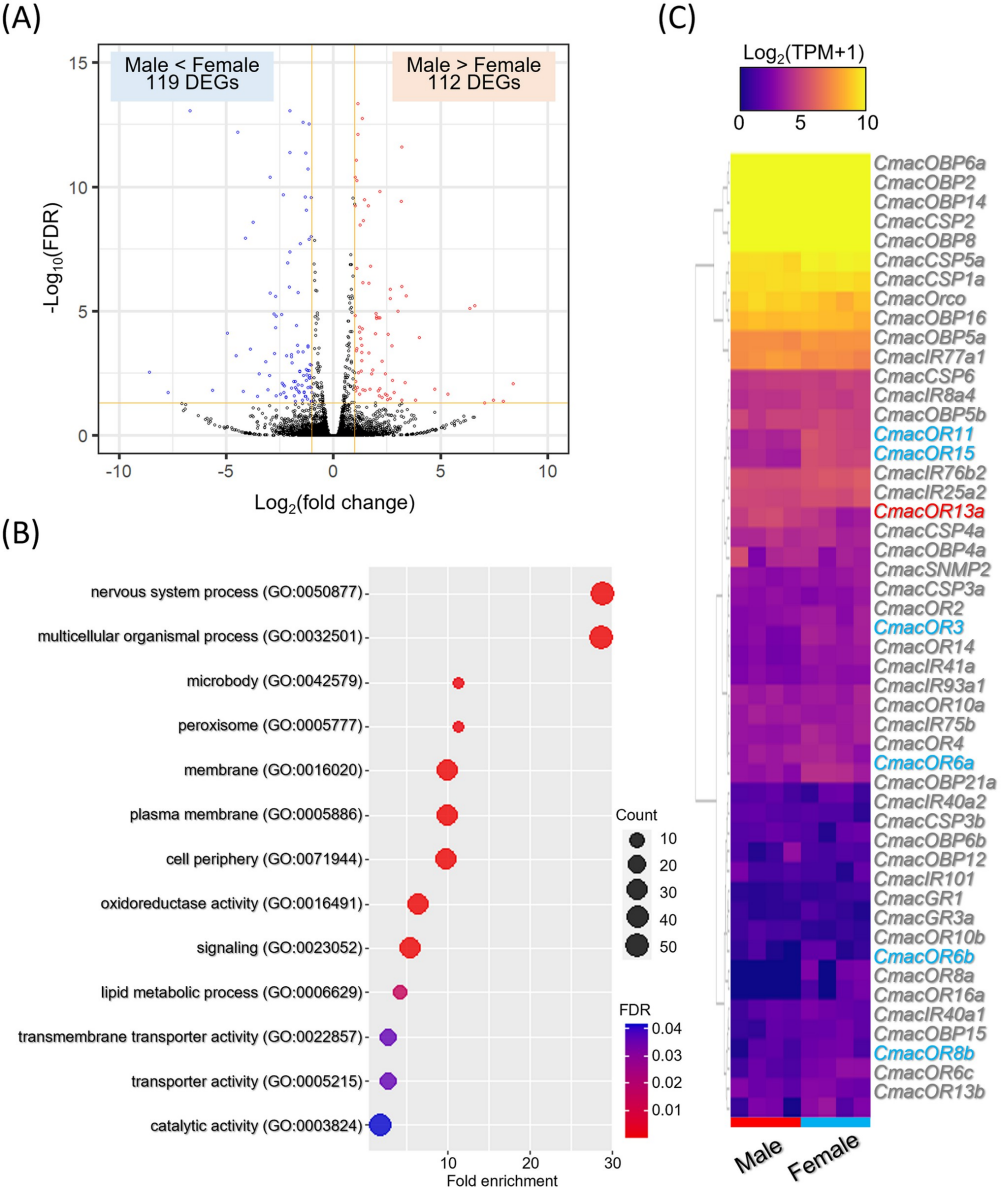
Differential gene expression between antennae of virgin males and females. (A) Volcano plot showing log2 fold change (FC) (*x*-axis) and log10 false discovery rate (FDR) (*y*-axis) plots of all expressed unigenes. The red plots represent the 112 upregulated DEGs (males > females) (FC ≥ 2 and FDR < 0.05), whereas the blue plots represent the 119 downregulated DEGs (males < females) (FC ≤ 2 and FDR < 0.05). (B) Dot plot showing fold enrichment, gene scale, and FDR derived from GO enrichment analysis. (C) Heatmap showing the 49 olfactory-related chemosensory gene expression profiling through *de novo* antennal RNA-seq. CmacOR13a in red and the six CmacORs in blue are shown as up- and downregulated DEGs, respectively.

**Fig 8:**
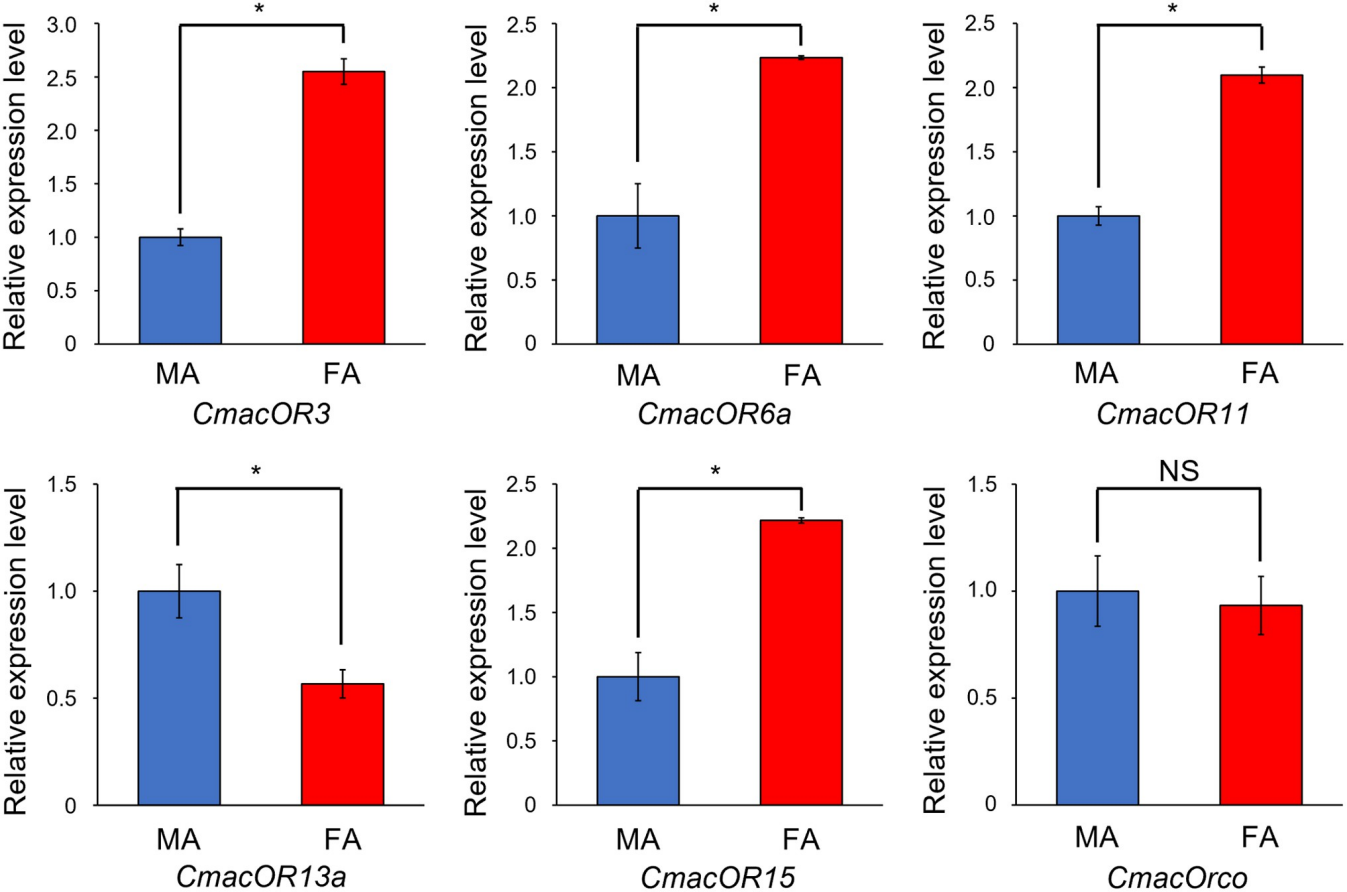
Sex-specific expression of *Callosobruchus maculatus* candidate OR and Orco genes using qRT-PCR analysis. MA, male antennae; FA, female antennae. Asterisks above the bars indicate a significant difference (*p* < 0.05, *t*-test); NS, no significant difference.

Using gene expression analysis of the reads mapped to the *de novo* assembled unigene sequences, 231 DEGs were detected between virgin male and female antennae, of which 112 upregulated and 119 downregulated genes were observed based on male antennae (Fig 7A). The GO enrichment analysis indicated that the DEGs were significantly enriched in four biological processes ("nervous system process," "multicellular organismal process," "signaling," and "lipid metabolic process"), five cellular components ("plasma membrane," "membrane," "cell periphery," "microbody," and "peroxisome"), and four molecular functions ("oxidoreductase activity," "transmembrane transporter activity," "transporter activity," and "catalytic activity") (Fig 7B). On the other hand, although the DEGs were identified in "cutin, suberin, and wax biosynthesis," "tryptophan metabolism," "folate biosynthesis," "histidine metabolism," and "one carbon pool by folate" based on 2-fold enrichment in the Kyoto Encyclopedia of Genes and Genomes pathway enrichment analysis, these pathways were not significantly enriched. Combining the DEGs with topology prediction, seven CmacORs revealed different expression patterns between sexes; CmacOR13a was upregulated while six CmacORs (CmacOR3, CmacOR6a, CmacOR6b, CmacOR8b, CmacOR11, and CmacOR15) were downregulated in male antennae (Fig 7C, S2 Table). To validate the expression profiles of CmacORs between virgin male and female antennae, we performed qRT-PCR for the five CmacORs, except for CmacOR6b and CmacOR8b, which have been suggested to be isoforms, and CmacOrco, which formed heteromeric complexes with all ORs. The analysis revealed that CmacOR13a was significantly upregulated in male antennae, whereas CmacOR3, CmacOR6a, CmacOR11, and CmacOR15 were significantly upregulated in female antennae (Fig 8). However, CmacOrco expression did not differ between the antennae of males and females.

## Discussion

Recent RNA-seq (transcriptome) analyses, especially of non-model organisms, have revealed continuous progress in high-throughput sequencing technology [69–71]. Therefore, in the present study, we used next-generation sequencing to analyze the antennal transcriptome of a non-model bean beetle, *C*. *maculatus*, and accessorily annotated predicted genes from its genomic data.

Olfactory-related chemosensory genes have been suggested as potential targets for pest management. Since there are limited coleopteran databases and limited annotation information on *C*. *maculatus*, we searched for unigenes and CDSs built from the genomic and amino acid sequences against the customized NCBI protein database. Based on the highest BLAST scores, we identified 99 candidate chemosensory genes, including 26 *ORs*, 9 *GRs*, 24 *IRs*, 1 *SNMP*, 22 *OBPs*, and 10 *CSPs*, which were verified using the transmembrane hidden Markov model, signal peptide predictions, and motif analyses. Of these genes, 17 *ORs*, 2 *GRs*, 10 *IRs*, 1 *SNMP*, 12 *OBPs*, and 7 *CSPs* were newly identified using *de novo* antennal RNA-seq.

In the insect olfactory system, OR forms heteromeric complexes with Orco. Although low-level amino-acid identities are shared between ORs, Orco sequences are highly conserved across distinct insect lineages [72]. In the phylogenetic analysis, CmacOrco revealed high similarity with three coleopteran Orco genes. The bulk of CmacORs was grouped in group 2A, followed by group 5A; 13 CmacORs formed a unique cluster in group 2A, indicating species-specific OR expansion (Fig 1). In Coleoptera, some species lost many OR subfamilies during evolution, and the ORs revealed a systematic lineage-specific expansion pattern following gain and loss [54]. Cucujiformia beetle genomic analysis revealed the expansion of subgroups 2A and 5A [54, 73], consistent with the present results.

The gustatory receptors of coleopteran beetles follow a common insect pattern; they include a CO_2_ receptor; putative sugar receptors, including those specific to fructose detection; and the remaining GRs are classified as putative bitter receptors assumed to be homologous with those in *Drosophila melanogaster* [73–76]. In the present analysis, no CO_2_ receptor was identified, and only sugar, fructose, and bitter receptors were suggested. Since TPM values of CmacGR1 and CmacGR3a derived from the antennal transcriptome were low (S3 Table), the GR expression was suggested to be low in the antennae.

Insect IRs are derived from ionotropic glutamate receptors and are divided loosely into antennal and divergent IRs; the antennal IRs show olfactory detection of acids and amines. Conversely, divergent IRs are associated with the gustatory function [6, 77]. In antennal transcriptome analysis, only one divergent CmacIR101 with low TPM values was detected (S4 Table); however, nine antennal CmacIRs with adequate TPM values were detected, suggesting that divergent IR expression could be low in the antennae. The IR75 beetle clade includes DmelIR75a–d and DmelIR64a homologs, which tend to radiate, ranging from 4 to 11 genes [55]. Furthermore, three CmacIRs within the IR75 clade were identified.

We identified only one SNMP gene cluster within the SNMP2 clade. Members of the SNMP1 subgroup are usually expressed in pheromone-sensitive olfactory neurons and are required for pheromonal activity in *Drosophila* flies and moths [78–82]. However, coleopteran beetle SNMPs remain poorly understood regarding whether the SNMPs could mediate pheromone reception, which requires further investigation.

Binding proteins (OBPs and CSPs) are involved in the first step of chemoreception in the lymph. On comparing the OBPs and CSPs between the congeneric beetles *C*. *maculatus* and *C*. *chinensis*, it was observed that three CmacOBPs and six CchiOBPs did not generate pairs (Fig 5). The sex attractant pheromone structures differed in both species; *C*. *maculatus* uses five short-chain fatty acids, whereas *C*. *chinensis* uses homosesquiterpene aldehydes [83], indicating saltational evolution of the structures [84]. Therefore, such unique OBPs may be involved in detecting the structurally different sex attractant pheromones.

Within Coleoptera, CSPs are highly conserved and expressed in many parts of beetles [55]. In the case of the Chinese white pine beetle, *Dendroctonus armandi* Tsai and Li, the DarmCSP2 involves not only carrying of pheromones but also various host plant volatiles [85]. Thus, CmacCSPs identified from the antenna-transcriptome might carry some semiochemicals.

Since adult *C*. *maculatus* do not feed, the most important chemosensory signals are for mating and host recognition for oviposition. qRT-PCR analyses confirmed that the expression pattern of CmacOrco between the antennae of males and females was not significant, and the same pattern was observed in other coleopteran species [30, 33, 35]. In honey bees, the male-biased expression OR is associated with detecting female-produced sex pheromones [86]. CmacOR13a is highly expressed in the male antennae, suggesting it might be related to sex pheromone reception. In contrast, CmacOR3, CmacOR6a, CmacOR11, and CmacOR15 were significantly more expressed in female antennae; these CmacORs might be related to identifying host legumes for oviposition.

In the present study, antenna-transcriptome-based chemosensory gene analyses suggested the presence of several chemosensory genes. Further functional analyses of chemosensory genes could facilitate the development of sustainable pest control strategies for *C*. *maculatus*.

## Supporting information

S1 Table Primers used in the qRT-PCR analysis. (XLSX)

S2 Table Candidate odorant receptor (OR) in *Callosobruchus maculatus* identified from the *de novo* antennal transcriptome and previous genomic construction data. (XLSX)

S3 Table Candidate gustatory receptor (GR) in *Callosobruchus maculatus* identified from the *de novo* antennal transcriptome and previous genomic construction data. (XLSX)

S4 Table Candidate ionotropic receptor (IR) in *Callosobruchus maculatus* identified from the *de novo* antennal transcriptome and previous genomic construction data. (XLSX)

S5 Table Candidate sensory neuron membrane protein (SNMP) in *Callosobruchus maculatus* identified from the *de novo* antennal transcriptome data. (XLSX)

S6 Table Candidate odorant-binding protein (OBP) in *Callosobruchus maculatus* identified from the *de novo* antennal transcriptome and previous genomic construction data. (XLSX)

S7 Table Candidate chemosensory protein (CSP) in *Callosobruchus maculatus* identified from the *de novo* antennal transcriptome and previous genomic construction data. (XLSX)
